# Acceptance and Commitment Therapy Wellness Program for Latine Adults Who Smoke and Have Psychological Distress: Protocol for a Feasibility Study

**DOI:** 10.2196/44146

**Published:** 2023-04-04

**Authors:** Virmarie Correa-Fernández, Janice A Blalock, Megan E Piper, Glorisa Canino, David W Wetter

**Affiliations:** 1 Department of Psychological, Health, & Learning Sciences University of Houston Houston, TX United States; 2 Department of Behavioral Science University of Texas MD Anderson Cancer Center Houston, TX United States; 3 Department of Medicine, School of Medicine and Public Health University of Wisconsin Madison, WI United States; 4 Behavioral Sciences Research Institute University of Puerto Rico Medical Sciences Campus San Juan Puerto Rico; 5 Huntsman Cancer Institute and the Department of Population Health Sciences University of Utah Salt Lake City, UT United States

**Keywords:** acceptance and commitment therapy, Hispanic or Latine, smoking, telehealth

## Abstract

**Background:**

Tobacco smoking is a major independent risk factor for chronic disease, and the prevalence of smoking among people with behavioral health disorders is 2-fold in comparison with the general population. Smoking rates remain high for various subgroups within the Latine community, the largest ethnic minority group in the United States. Acceptance and commitment therapy (ACT) is a theoretically sound and clinically validated therapeutic approach for several behavioral health conditions with growing evidence of its effectiveness for smoking cessation. Unfortunately, the evidence of ACT effectiveness for smoking cessation among Latine individuals is scarce, and none of the existing studies have tested a culturally targeted intervention for this population.

**Objective:**

This study aims to address the co-occurrence of smoking and mood-related challenges among Latine adults via the development and testing of a culturally tailored ACT-based wellness program: Project PRESENT.

**Methods:**

This study entails 2 phases. Phase 1 consists of the intervention development. Phase 2 entails the pilot testing of the behavioral intervention along with the administration of baseline and follow-up measures to 38 participants. Primary outcomes include feasibility of recruitment and retention, and treatment acceptability. Secondary outcomes are smoking status and depression and anxiety scores at end of treatment and 1-month follow-up.

**Results:**

This study received institutional review board approval. Phase 1 outputs were the health counselors’ treatment manual and participant guide. Recruitment was completed in 2021. Phase 2 outcomes will be determined after project implementation and data analyses are complete, which are expected by May 2023.

**Conclusions:**

Findings from this study will determine the feasibility and acceptability of an ACT-based, culturally relevant intervention for Latine adults who smoke and have probable depression and/or anxiety. We expect feasibility of recruitment, retention and treatment acceptability, and reductions in smoking status, depression, and anxiety. If feasible and acceptable, the study will inform large-scale trials, which will ultimately contribute to narrowing the gap between research and clinical practice for the co-occurrence of smoking and psychological distress among Latine adults.

**International Registered Report Identifier (IRRID):**

DERR1-10.2196/44146

## Introduction

Tobacco smoking is a major independent risk factor for chronic disease [1,2]. Although the number of smokers in the United States has declined over the past decades, smoking prevalence remains disproportionately high among individuals with behavioral health disorders [3-5]. For instance, depressive and anxiety syndromes are more prevalent among smokers than nonsmokers [6,7], and smokers with these conditions are more likely to be nicotine dependent [4,8]. Considerable research has assessed the impact of depression and anxiety on cessation outcomes, with a number of studies reporting that depression and anxiety place smokers at increased risk for cessation failure [9-14], with other studies suggesting that the evidence is inconclusive [6,15-18]. Additionally, given the high comorbidity between depression and anxiety [19], the independent contribution of each disorder to smoking cessation remains unclear [11,20,21]. The *Treating Tobacco Use and Dependence Clinical Practice Guideline: 2008 Update* concluded that the evidence is insufficient to determine whether smokers with psychiatric disorders benefit more from tobacco use treatment tailored to their disorder than from standard treatments [22].

Given the link between behavioral health issues and smoking, it would be ideal to identify an intervention that might be especially appropriate for people with behavioral health issues who smoke. Acceptance and Commitment Therapy (ACT) belongs to what is known as “third wave” cognitive behavioral therapy and is positioned as a form of “contextual cognitive behavioral therapy” [23]. An important and unique assumption underlying ACT’s treatment approach is that, through conditioning processes, humans learn to avoid thoughts and their accompanying feelings, images, and physical sensations as they would avoid the event itself [24]. This avoidance of aversive private events, called experiential avoidance, is assumed to underlie much of people’s suffering, and emotional dysregulation arises from attempts to avoid and alter private experiences that are judged to be aversive [25].

Unfortunately, experiential avoidance often results in an increase in the frequency or intensity of the avoided thoughts and feelings [26] and can generate mood disturbances, including depression and anxiety. An important component of ACT is helping individuals abandon efforts to change their thoughts and feelings and instead engage in an active process of experiencing emotions simply as a constellation of physiological sensations, which have no intrinsic power to harm or hold one back. The goal is the removal of experiential avoidance as a barrier to pursuing valued outcomes (ie, quitting smoking and improving mood).

With respect to substance abuse, behaviors like tobacco smoking are often negatively reinforced via attempts to regulate and control internal, negative experiences [27]. In ACT-based models for the treatment of tobacco use disorders [28], individuals are helped to identify subtle signals of negative affect and to understand that efforts to control or avoid internal experiences are linked to their tobacco use behavior. They are then taught to develop acceptance and willingness to remain in the presence of withdrawal symptoms and aversive internal states associated with triggers to smoke tobacco.

ACT has increasingly shown its effectiveness for treating smoking, depression, and anxiety [23,29-31], and its applicability to diverse populations [32]. However, research on the usefulness of ACT approaches for Hispanic or Latine (hereafter *Latine*, a gender-neutral term) populations, the largest ethnic minority group in the United States [33,34], is limited. Although there are some ACT-related studies that include a considerable proportion of Latine people in their samples [29,35], there is only 1 published study specifically focused on Latine individuals in the United States [36]. However, this study is a secondary data analyses of treatment engagement and efficacy from an ACT treatment not culturally tailored to the population. Moreover, no published studies have specifically addressed smoking cessation among Latine individuals with depressive and anxiety symptomatology.

These gaps in the literature illustrate the need for an ACT smoking cessation treatment that is appropriate for Latine individuals with behavioral health issues. ACT represents a coherent theoretical framework from which to address many of the factors that may present barriers to smoking cessation among Latine individuals with depression and anxiety symptomatology. Hence, the proposed study (ie, PRESENT Wellness Program) aims to improve the health of the Latine community by developing and pilot-testing a culturally relevant ACT-based wellness program addressing smoking, depression, and anxiety among Latine adults.

## Methods

### Phase 1: Intervention Development: The PRESENT Wellness Program

The PRESENT Wellness Program consists of the ACT-based behavioral treatment component with a participant’s guide and nicotine replacement therapy (NRT).

#### Behavioral Treatment Component and Participant Guide

The techniques used in ACT target the following six main processes [37]: (1) *acceptance*—willingness to experience the natural flow of thoughts, feelings, and sensations (not trying to suppress them); (2) *cognitive defusion—*ability to recognize thoughts and images as just words and pictures, not as real events; (3) *being* *present—*focus on the present in a nonjudgmental way; (4) *self-as context—*exposure to experiential processes to promote awareness of one’s own flow of experiences without attachment to them; (5) *values—*awareness and clarification of personal values and goals; and (6) *committed action—*willingness to behave in line with values and goals, even in the presence of discomfort.

The baseline behavioral treatment was developed using an ACT-based smoking cessation protocol described in existing publications on ACT and smoking cessation [38-40]. This baseline treatment protocol was then adapted to also address depression and anxiety and to be culturally appropriate for Latine adults. The behavioral health and cultural adaptations were informed by an existing framework for adaptation [41,42], the first author’s training on the ACT therapeutic model, and expert consultation. A literature review informed changes to content and to the frequency and duration of sessions.

Adaptations of the baseline protocol to address co-occurring depression and anxiety entailed the proactive inclusion of emotional dysregulation discussions and experiences as part of the treatment. Regardless of participants’ initiative to share their experiences with mood management, the health counselor proactively inquires about the person’s psychological distress, validates their pain, and highlights that facing discomfort is an inevitable human experience oftentimes necessary for valued living. ACT works to enhance an individual’s ability and willingness to experience undesirable or adverse thoughts and feelings, which is expected to assist individuals in developing broader alternatives to mood management. Adaptations of the baseline protocol to be culturally appropriate for Latine individuals started with the labeling of the treatment. For instance, given Latine individuals’ stigma related to seeking behavioral health help [43], we chose to call the treatment a “wellness program” so it could be better received by the Latine community. Similarly, we use the terms “health counselor” and “health counseling” to refer to the therapist and the treatment sessions, respectively, to increase openness to the program. Adaptations were also based on the ideas of cross-cultural communication [44,45] as well as the integration of Latine values (eg, familism, personalism, and collectivism) and context (eg, heritage group, acculturation, and enculturation) [46].

The treatment manual includes overall training modules that cover the ACT model, tobacco dependence, depression, anxiety, Latine cultural values, and a session-by-session guideline that includes the core aspects of ACT and their applications for treatment of tobacco dependence and depression or anxiety. The participant’s guide mirrored the counselors’ treatment manual content in a more simplified manner and includes practice exercises.

The treatment protocol consists of 8 one-hour individual sessions. *Session 1* entails the contextual interview (focused on smoking and psychological distress) and offers didactic information about the effectiveness and use of NRT. The contextual interview is organized around valued domains of living (ie, relationships, health, work, education, and leisure) and the functionality of smoking. Sessions 2-7 focus on each one of the 6 core ACT processes [23,37]. *Session 2* focuses on clarifying participants’ overall values and those involved in quitting smoking, as well as the discrepancies between current behaviors and values. *Session 3* focuses on discussing and demonstrating what SMART goals are (ie, specific, meaningful, adaptive, realistic, and timebound) and the development of a preliminary plan to stop smoking, including setting a quit date. *Session 4* introduces participants to mindfulness and helps them notice their internal and external triggers as they occur and to recognize the link between connecting with emotions and the ability to act on them. *Session 5* aims to increase participants’ willingness to accept cravings and withdrawal symptoms as a typical experience of the quitting process and develop motivation and skills to deal with them without smoking. *Session 6* helps participants identify and defuse (or to get “unhooked”) from thoughts that limit their achievement of quitting smoking and mood-related behavioral goals. In this context, defusion is to see thoughts simply as thoughts rather than as a literal truth that can control one’s behavior. *Session 7* guides participants to connect with a transcendent sense of self that is separate from their own internal experience but provides a safe place to observe them. *Session 8* focuses on overall experiences during the wellness program as well as the creation of an individual self-care plan for the future, including value-based actions. All sessions include experiential exercises and metaphors tailored to the participant’s situation. Latine-specific issues are incorporated into each session. Participants are instructed to engage in value-based actions as homework assignments between sessions. Table 1 below shows a summary of the session-by-session content with focal exercises [47].

**Table 1:** Summary of session-by-session content.

Session	Overview of the session	Experiential exercise
1	Contextual interview, explanation of the ACT^a^ model and overall wellness program, and didactic information about NRT^b^	Life path
2	Values identification	Bull’s eye
3	Goal setting and committed action (quit day is encouraged)	Establishing SMART^c^ goals
4	Contact with the present moment	Mindful breathing
5	Acceptance and willingness	The ball in the pool metaphor
6	Cognitive defusion (getting unhooked!)	Hands as thoughts metaphor
7	Self as context (also called perspective taking)	The stage show metaphor
8	Integration and maintenance	Miracle question and Passenger on the bus metaphor

^a^ACT: acceptance and commitment therapy.

^b^NRT: nicotine replacement therapy.

^c^SMART: specific, meaningful, adaptive, realistic, and timebound.

Health counselors with at least a master level degree in clinical or counseling psychology are trained to provide the PRESENT Wellness Program. Specifically, the health counselors undergo about 100 hours of specialized training in tobacco dependence treatment and ACT, including didactic, experiential, and applied training. Before engaging with study participants, health counselors role-play the treatment sessions and have to demonstrate ACT competency, measured by the ACT Core Competency Rating Form [37].

#### Guideline for NRT Use

NRT is offered as part of the PRESENT Wellness Program. The selected NRT is the nicotine patch because it is frontline therapy for smoking cessation, is safe, well-tolerated, and available over the counter [22]. Also, the nicotine patch has proven effective among smokers with depression and anxiety symptomatology [48]. Participants are given 6 weeks of nicotine patches and are told to start using them 1 week before the quit day, which is encouraged on Session 3. Participants smoking more than 10 cigarettes per day are given 4 weeks of 21-mg patches, 1 week of 14-mg patches, and 1 week of 7-mg patches. Participants who smoke 5-10 cigarettes per day are given 4 weeks of 14 mg patches and 2 weeks of 7 mg patches.

### Phase 2: Pilot Testing: Study Design and Participants

This is a longitudinal, 1-arm pre-post feasibility study. A total of 38 participants will be enrolled in the study. The inclusion criteria are ≥18 years of age; self-identify as Latine (of any national group); current smoker (average of >5 cigarettes per day for the past year and carbon monoxide [CO] >6 ppm); motivated to quit within next 30 days; screened positive for probable depression and/or probable anxiety (via a score of >10 in the Patient Health Questionnaire [49-51]; at least marginal health literacy; functioning telephone number; ability to speak English; and physicians’ release to participate if taking psychotropic medications. Of note, we chose to recruit only English-speaking Latine participants because it facilitates the supervision of sessions and preparation of materials in only 1 language, maximizing the feasibility of completing the study within the time frame and available resources. The exclusion criteria are contraindication for use of nicotine patch; current use of tobacco cessation medications; current participation in counseling for depression, anxiety, or smoking cessation; being pregnant or nursing; having other current psychiatric disorder that would limit ability to participate; and having a household member enrolled in the study.

### Procedures

#### Recruitment and Screening

Participants are mainly recruited through community outreach, social and print media (eg, local newspaper), flyers, and the ResearchMatch platform [52]. To determine eligibility criteria, interested people complete a phone screening with project staff or complete a self-screening accessed via a QR code located in the study flyer. Individuals who are ineligible or decline participation are given self-help materials and referrals to other cessation programs. Eligible individuals are scheduled for their first appointment.

#### Baseline Visit

During the in-person baseline visit, study personnel provide a detailed description of the study, answer questions, and obtain informed consent. Enrolled participants complete web-based baseline questionnaires, biochemical verification of smoking status (via a CO test), and their first health counseling session.

#### Treatment Procedures and Fidelity

The health counseling component consists of 8 one-hour individual sessions (1 in person and 7 by phone) completed within a 3-month period. The initial 2-week nicotine patch is dispensed at the first face-to-face visit and then sent biweekly by regular mail. Participants receive only the number of patches necessary to last 2 weeks, plus several extra patches should a patch fall off or become torn.

Treatment fidelity refers to the extent to which an intervention is delivered as planned. To ensure treatment fidelity, all sessions are recorded and a random sample of at least 15% are coded using the following two forms: (1) an investigator-developed checklist to track fidelity to treatment content per session and (2) the ACT Core Competency Rating Form to determine adherence to an ACT therapeutic approach [37]. The checklist entails a list of all session components (per session), which are marked as covered or not covered. The rating is calculated by assigning 10 points to each component marked as covered and dividing by the maximum number of components in that particular session (see the Multimedia Appendix 1). In the ACT Core Competency Rating Form, the rater evaluates the counselor’s competency in ACT on a scale of 1-7, from “never true” to “always true.” The supervisor provides written feedback based on the ratings of the rated sessions. Low treatment adherence is addressed by additional training and repeated assessment of adherence on additional cases. Treatment fidelity is attained when 85% or more (ie, ≥8) of the session components were covered and when receiving an overall rating of adequate or above adequate delivery (eg, ≥5) of the ACT essential components.

#### Follow-up Assessments and Treatment Evaluation

After the final health counseling session, participants attend 2 in-person follow-up visits, at 1-week and 1-month posttreatment, in which they complete a CO test as well as web-based questionnaires about tobacco use, depression, anxiety, and ACT-related constructs. At the 1-month visit, participants rate the degree of acceptability and helpfulness of each ACT component and their satisfaction with the PRESENT Wellness Program.

#### Financial Compensation

Participants are compensated in the form of gift cards for each of the in-person visits in which they complete assessments (ie, US $30 for baseline and 1 week after end of treatment; and US $40 for 1-month posttreatment follow-up). Thus, participants could receive a maximum total of US $100/person for the completion of assessments and health counseling sessions.

### Measures

#### Primary Outcomes

The primary outcomes of the study are feasibility of recruitment, feasibility of retention, and treatment acceptability. The feasibility of recruitment will be measured by the proportion of participants who consented to participate and attended the baseline session out of the number of individuals who were eligible. The feasibility of retention will be measured by the number of health sessions completed and the rate of follow-up visit completion. Treatment acceptability will be measured by the Program Acceptability Questions (see the Multimedia Appendix 2), an investigator-developed questionnaire rating the degree of acceptability and helpfulness of the PRESENT Wellness Program. The questionnaire contains 7 items categorized on a 5-point Likert scale ranging from “Completely Disagree” to “Completely Agree.” Higher scores indicate higher acceptability. A sample item is “This program has helped in my acceptance of my physical cravings, emotions, and thoughts that cue my smoking.”

#### Secondary Outcomes

##### Smoking Abstinence

Seven-day point prevalence and continuous abstinence will be reported following the Society for Research on Nicotine and Tobacco guidelines [53,54]. *Seven-day point prevalence* abstinence is defined as a self-report of no smoking during the previous 7 days and a CO level of less than 6 ppm. *Continuous abstinence* is defined as a self-report of no smoking since the quit date and biochemically confirmed abstinence at all follow-ups up to and including that time point.

##### Probable Depression

Probable depression is measured by the Patient Health Questionnaire [49,50], which evaluates symptoms for the last 2 weeks. Items range from not at all (0) to nearly every day (3). The scores for each item are summed to produce a total score between 0 and 24 points. Higher scores indicate higher depressive symptoms. Probable depression is determined by a total score of 10 or above, indicating at least moderate symptoms [50].

##### Probable Anxiety

Probable anxiety is measured by the Generalized Anxiety Disorder Scale [51], which evaluates symptoms for the last 2 weeks. Items range from not at all (0) to nearly every day (3). The scores for each item are summed to produce a total score between 0 and 21 points. Higher scores indicate higher anxiety symptoms. Probable anxiety is determined by a total score of 10 or above, indicating at least moderate symptoms [51].

#### Data Analyses

Data analyses in the context of a pilot study are not hypothesis-driven but serve to provide information regarding feasibility and acceptability of the intervention [55]. As such, data analyses will include descriptive statistics (eg, mean tests and proportions) about screening, recruitment, retention, process assessments, and treatment acceptability. We will use descriptive analysis techniques to present participants’ characteristics, as well as their scores on secondary outcome measures at the 3 different time points (baseline, 1 week after end of treatment, and 1-month posttreatment).

### Ethics Approval

The first author and principal investigator of the study received institutional review board approval from her academic institution, the University of Houston (#00001007).

## Results

The outputs of the intervention development (phase 1) were (1) a treatment manual, which guided the health counselors’ training, and (2) the participant’s guide. Recruitment was completed in 2021. After project implementation and data management and analyses are conducted, the outcomes of the pilot study (phase 2) will be published in a separate paper. Dissemination of outcomes from phase 2 is slated for May 2023.

## Discussion

Despite the availability of evidence-based interventions for smoking cessation, there is still a need for interventions focused on individuals with behavioral health challenges, given their high smoking rate and difficulty quitting. Similarly, culturally appropriate interventions for the Latine community, the largest ethnic group in the United States, are greatly needed. To address these gaps, the innovation of the PRESENT Wellness Program lies in the adaptation and testing of an ACT smoking cessation intervention for Latine smokers with clinically significant depression and anxiety symptomatology. This entails refinements and new applications of an existing theoretical and evidence-based clinical approach (ie, ACT) for understudied comorbid conditions (ie, depression, anxiety, and smoking) among an underserved and understudied ethnic minority group (ie, Latine). Besides adaptations in content, the proposed protocol is delivered in a hybrid format, with 1 session in person and the rest by phone, which is expected to address common treatment barriers for Latine individuals (eg, childcare and transportation). All these aspects are strengths of the study.

The implementation of the PRESENT Wellness Program will provide data on the feasibility and acceptability of the intervention as well as preliminary data on the impact of the intervention on decreasing smoking, anxiety and depression, and addressing ACT-related constructs. As primary outcomes, it is expected that recruitment and retention into the intervention is feasible and that the provided treatment is acceptable. For secondary outcomes, it is expected that there is an increase in participants’ smoking abstinence and a decrease in depression and anxiety symptoms at the end of treatment and 1-month posttreatment. Our findings will help inform large-scale ACT-based smoking cessation studies for Latine adults with psychological distress and may contribute to a growing body of evidence of the importance of culturally relevant interventions. Findings will be disseminated via professional forums as well as community-accessible venues, such as our laboratory website, social media, and newsletters.

Of note, this pilot study was focused on English-speaking Latine individuals mainly for pragmatic reasons related to study budget and the feasibility of conducting the intervention and related supervision in only one language. Nonetheless, we acknowledge this is a limitation as excluding Spanish-preferring individuals will reduce the generalizability of findings and the reach of the intervention. We believe there is an urgency to reach Spanish-speaking Latine individuals who smoke and, as such, will work on the translation and linguistic adaptation of the intervention as a next step in this line of research.

## Appendix

Multimedia Appendix 1 Treatment Fidelity: Checklist Sample.

Multimedia Appendix 2 Program Acceptability Questions.

## Abbreviations

ACT: acceptance and commitment therapy
CO: carbon monoxide
NRT: nicotine replacement therapy
SMART: specific, meaningful, adaptive, realistic, and timebound

## Data Availability

Data sharing is not applicable to this research protocol paper as no clean data sets have been generated for data management or analyses in this study.
