# Supplementary material for: Acceptance and Commitment Therapy Wellness Program for Latine Adults Who Smoke and Have Psychological Distress: Protocol for a Feasibility Study
Source: JMIR Res Protoc. 2023 Apr 4;12:e44146. doi: 10.2196/44146 (PMC10131986; doi:10.2196/44146)
Supplement: Multimedia Appendix 1 [file resprot_v12i1e44146_app1.docx]

**PRESENT Wellness Program**

**Treatment Fidelity Form Sample**

| **Covered** | **Not Covered** | **Session 3 components** |
| --- | --- | --- |
|  |  | Check-in about “at-home” skill practice and experience between sessions |
|  |  | Discussion of what a S.M.A.R.T goal is and set S.M.A.R.T goals in relation to smoking |
|  |  | Encourage participant to select a quit day |
|  |  | Social support and plan to notify close network about quitting |
|  |  | Determine a skill practice and inquire readiness to perform it |
|  |  | Quick evaluation of session and scheduling of next session |

6 components: 10 points each “Covered”

Rating: # of “covered” x 10 divided by # of components in this session

Developed by:

*Virmarie Correa-Fernández, Ph.D. and the Latino and Behavioral Health Research Team* *for the evaluation of the PRESENT Wellness Program, a pilot study.* [Funded by the American Cancer Society; MRSG-15-018-01-CPPB]
