# Supplementary material for: Acceptance and Commitment Therapy Wellness Program for Latine Adults Who Smoke and Have Psychological Distress: Protocol for a Feasibility Study
Source: JMIR Res Protoc. 2023 Apr 4;12:e44146. doi: 10.2196/44146 (PMC10131986; doi:10.2196/44146)
Supplement: Multimedia Appendix 2 [file resprot_v12i1e44146_app2.docx]

**PRESENT Wellness Program**

**Program Acceptability Questions**

*Rate the following questions on a scale from 1-5, with 1 =“completely disagree”, 2= “somewhat disagree”, 3= “neutral”, 4= somewhat agree, and 5= “completely agree”*

1. Improvements in my mood in the past month have helped to quit smoking.
2. Decreases in my smoking behavior in the past month have improved my mood.
3. This program has positively impacted my smoking behavior.
4. This program has helped in my acceptance of my physical cravings, emotions, and thoughts that cue my smoking.
5. The experience with my program health counselor was positive.
6. My experience with an over the phone smoking cessation program was positive.
7. This program was applicable to someone such as myself who identifies with a Hispanic/Latino background.

Developed by:

*Virmarie Correa-Fernández, Ph.D. and the Latino and Behavioral Health Research Team for the evaluation of the PRESENT Wellness Program, a pilot study.* [Funded by the American Cancer Society; MRSG-15-018-01-CPPB]
